# Supplementary material for: Are carbon emissions trading and green financial instruments synergistic? -Comprehensive quantitative research based on content analysis
Source: PLoS One. 2024 Mar 7;19(3):e0298601. doi: 10.1371/journal.pone.0298601 (PMC10919601; doi:10.1371/journal.pone.0298601)
Supplement: S1 Data — (ZIP) [file pone.0298601.s001.zip › Data/地级市Z_F工作报告.zip!/╒π╜¡/╬┬╓▌╩╨/└┤╫╘╣1⁄2╓┌║┼í╛┬φ┐╦╩2╛▌═°í┐_2018.docx]

各位代表：

现在，我代表温州市人民政府向大会报告政府工作，请予审议，并请各位政协委员和其他列席人员提出意见。

一、2017年工作回顾

2017年是本届政府的开局之年。我们认真学习贯彻习近平新时代中国特色社会主义思想，在中共温州市委的坚强领导下，围绕系统构建五大发展生态、建设国际时尚智城、打造坚实“铁三角”，立足全局、谋划开局、力创新局，较好地完成了市十三届人大一次会议确定的主要目标任务。全年实现地区生产总值5453.2亿元，增长8.4%，增速连续三年居全省前三位；一般公共预算收入465.4亿元，增长7.2%；城乡居民人均可支配收入分别为51866元和25154元，分别增长8.5%和9.4%，城乡居民收入比进一步缩小。

一年来，我们紧紧抓住供给侧结构性改革这条主线，着力优化经济结构，促进新旧动能加快转换。坚持创新驱动、人才引领，国家自主创新示范区获批，国家重点实验室建设实现零的突破，全市新增高新技术企业179家、科技型中小企业1095家、省级企业研究院26家。省级“千人计划”产业园落户浙南科技城，全市新增国家级重大人才工程人选12人、省“千人计划”23人，入选人数均创历史新高。坚持两化融合、智能制造，力推传统产业改造提升，培育两化融合示范试点企业28家、智能制造示范企业16家、“上云”企业1.5万家，推广机器人应用1215台。新增“浙江制造”标准14个、“浙江制造”品牌认证6个。坚持平台带动、集聚发展，瓯江口产业集聚区、浙南产业集聚区产城融合、项目招引取得积极成果。新增市级以上众创空间64家，新开工小微园23个、竣工368万平方米。坚持降本减负、培优扶强，推出企业上市“十条新政”，新增上市企业4家，形成超200家的拟上市企业梯队，省级“隐形冠军”培育企业数居全省第一。开展“十百千”助企服务活动，为企业减负122.4亿元，解决难题3820个。加大风险企业分类处置力度，银行贷款不良率降至1.92%。坚持产业融合、协调发展，高效生态现代农业发展势头良好，现代服务业增加值增长9.7%，全年接待游客数突破1亿人次，旅游总收入超1000亿元。

一年来，我们坚决打破坛坛罐罐，联动推进“大拆大整”“大建大美”，城乡环境面貌持续改善。完成城中村改造签约9.8万户、拆除8.6万户，其中整村签约85个、拆除83个。关停取缔“四无”生产经营单位5.3万家，整治旧市场140家，搬迁改造市区工业企业1249家，获评省“腾笼换鸟”先进市。5784幢城镇危旧房、3.8万户农村危旧房全面完成治理改造，城中村改造、危旧房治理和拆违工作考核均列全省第一。高起点谋划、实质性启动“大建大美”，开工建设学校、卫生、养老、道路等功能性项目195个，建成56个，“两线三片”开发建设兴起热潮，乐清、瑞安、平阳、苍南等城市新区建设提速。完成69条城市道路综合整治。开展城市管理治乱专项行动，城市视觉空间进一步美化。文成、永嘉、泰顺获评小城镇环境综合整治省级优秀县。建成美丽乡村风景线13条，创建3A级景区村25个，洞头创成省美丽乡村示范县，永嘉成为全国休闲农业和乡村旅游示范县。积极配合中央环保督察、国家海洋督察，集中解决了一批突出环境问题。全力打好劣V类水剿灭战，20个县控以上劣V类水质断面、2947个小微水体全部实现验收销号，基本实现农村生活污水治理设施长效运维管理。

一年来，我们狠抓重大项目谋划盯引，扩大有效投资取得积极进展。先后组织两批140个重大项目集中开工，固定资产投资增长11.9%。机场T2航站楼基本建成，绕城高速西南线全线通车，市域铁路S1线S2线、甬台温高速复线、龙丽温高速瑞文段等加速推进，杭温高铁一期顺利开工，完成交通投资351亿元，创历史新高。鳌江干流治理水头段、乐柳虹平原、瑞平平原等防洪排涝工程开工建设。完成17座城镇污水处理设施提标改造，市区西片污水处理厂二期扩建工程、西向生态填埋场建成投用。强化重大产业项目谋划盯引，招商引资到位资金696亿元，浙商回归考核列全省第一。新引进10亿元以上制造业项目9个，唯品会中国创新创业中心、青山瑞浦能源动力电池产业园、华峰新材料产业园等项目回归落地，重大产业项目投资增长25%。多渠道破解要素瓶颈制约，垦造耕地1.7万亩，实施城镇低效用地再开发3.4万亩；发行企业债39.2亿元，争取中央、省补助资金16.5亿元。

一年来，我们牢牢抓住“最多跑一次”改革这个“牛鼻子”，推动改革开放迈出新步伐。梳理公布1228项“最多跑一次”事项，推出10321个网上申办事项，政务服务网日均在线服务人次近2万人。推行“一窗受理、集成服务”，推出“四个一”创新工程，着力打破信息孤岛，审批提速率达51.2%，“最多跑一次”改革专项满意度评估居全省首位。深化金融综合改革，开展国家海域综合管理创新试点，全国“三位一体”新型农村合作体系建设现场会在我市召开。推动开放型经济加快发展，进出口总额增长11.2%，完成境外投资6.9亿美元，实际利用外资增长47.2%。开通温州港至东南亚集装箱航线，国际邮轮码头开港通航。航空口岸获批开展外国人口岸签证业务，新增国际(地区)航线3条。

一年来，我们坚守以人民为中心的发展思想，全面推进社会建设，群众的获得感进一步增强。坚持把全市三分之二以上的财政支出增量用于民生，省市民生实事项目全面完成。大力推进基础教育优质均衡发展，新改扩建中小学校162所、幼儿园94所，义务教育质量评价监测指标在全省晋等升位。支持温州医科大学创成省重点建设高校。全市新增骨干医院10家、住院床位7000张，国家卫生县城实现全覆盖。深化公立医院改革，全面建立分级诊疗制度，县域诊疗率达88%，基层就诊率达55%。新增文化礼堂165个、城市书房23家、文化驿站40家、百姓书屋22个。全国社会力量办体育试点落户温州，成功举办市第16届运动会，建成投用奥体中心一期，新增体育场地153万平方米，总面积跃居全省首位。全市户籍人口基本养老、医疗保险参保率分别达到85.9%、98.8%。最低工资标准和城乡居民最低生活保障标准进一步提高。完成低收入农户异地搬迁1.5万人，全面消除集体经济薄弱村1707个。全市所有建制村实现客车“村村通”。全国文明城市通过复评，移风易俗工作扎实推进，礼让斑马线蔚然成风，“兰小草”等一批“最美温州人”成为道德楷模。获得全国首批社会信用体系建设示范城市称号。工会、共青团、妇女儿童、老龄、慈善、红十字、残疾人等事业进一步发展，民族、宗教、双拥、对台、外事侨务等工作取得新进步。

一年来，我们加强和创新社会治理，深化平安建设，社会大局保持和谐稳定。圆满完成党的十九大、第四届世界互联网大会等维稳安保任务。成功化解信访积案1753件。扎实推进全科网格建设，镇街基层治理“四个平台”实现全覆盖。平稳完成村(居)、社区组织换届工作。加快构建立体化社会治安防控体系，“两抢”案件、命案发案数分别下降57.6%、20.3%。深化安全生产专项整治，各类事故起数下降31.4%，死亡人数下降29.7%。开展“除险安居”专项行动，消除地质灾害隐患点688个，新增避灾安置场所449个，2.3万人摆脱地质灾害威胁。完成农贸市场改造提升209家，农产品质量安全抽检合格率稳定在98%以上。

各位代表，过去一年我们紧紧围绕迎接十九大、学习贯彻十九大精神，切实加强政府自身建设，在学习型政府、服务型政府、责任政府、法治政府和廉洁政府打造上迈出了新步伐。围绕推动省委、省政府和市委重大决策部署的落实，实施政府工作“134”责任机制，年初确定的596项重点工作、1932条工作举措完成率达97.2%。主动接受市人大法律监督、工作监督和市政协民主监督，认真听取党外人士意见建议，办理人大代表建议623件、政协提案638件。严格落实中央八项规定精神，出台优化营商环境“八条禁令”，强化审计监督问责，正风肃纪常态化机制逐步形成。

各位代表，过去一年我们聚焦改革、聚焦发展、聚焦民生，在继承中创新、在发展中争先、在转型中突破，取得了来之不易的成绩，实现了本届政府的良好开局。这是习近平新时代中国特色社会主义思想和“八八战略”在温州生动实践的结果，是省委、省政府和市委正确领导的结果，是市人大、市政协有效监督和鼎力支持的结果，更是全市人民勠力同心、奋力拼搏的结果。在此，我代表市人民政府向全市人民，向人大代表、政协委员，各民主党派、工商联、人民团体和社会各界人士，向驻温部队、武警官兵和中央、省驻温单位，向投身温州建设的新老温州人、情系家乡的海内外温州人、关心支持温州发展的国内外友人，表示崇高的敬意和衷心的感谢！

我们也清醒地看到，当前温州发展中不平衡不充分的突出问题尚未得到有效解决，政府工作仍面临诸多困难和挑战。主要是：经济发展还没有摆脱传统路径依赖，发展质量不高，新旧动能转换接续不畅，一些指标在全省排位靠后，保持经济持续向好的压力较大。改革开放初期形成的体制机制优势逐渐消退，对外开放水平有待提高，重点领域改革攻坚力度亟需加强。都市区能级不高、集聚辐射能力不强，基础设施建设欠账较多，拆后利用速度不快，城市管理还不够精细。城乡和区域发展不协调的矛盾比较突出，低收入群众增收依然困难，实现高水平全面小康的任务紧迫。社会建设水平相对滞后，关系群众切身利益的优质教育、医疗、居住、养老等方面还有不少短板，生态保护和环境治理任务依然艰巨，社会文明水平尚需提升，维护社会安全稳定的压力不小，公共安全风险管控能力有待提高。政府自身建设还需加强，一些公务人员的思想观念、能力素质、担当精神还不适应新时代发展要求，改善营商环境、提升行政效能的呼声比较强烈。我们一定高度重视这些问题，以不畏艰难的勇气、克难攻坚的意志，采取有效措施努力加以解决。

二、2018年主要目标和重点工作

今年是全面贯彻党的十九大精神的开局之年，是改革开放40周年，是决胜高水平全面建成小康社会、实施“十三五”规划承上启下的关键一年。党的十九大全面开启了中国特色社会主义新时代，我国经济已由高速增长阶段转向高质量发展阶段，社会主要矛盾已转化为人民日益增长的美好生活需要和不平衡不充分的发展之间的矛盾。我们要主动适应新形势，在市委的坚强领导下，把握“五大历史性机遇”，破解“三道历史性命题”，切实把十九大提出的各项战略部署转化为新时代温州新发展的创造性实践，谋划实施一批最能激发活力的改革举措、最能带动全局的重大项目、最能提振信心的重点工作、最能补齐短板的民生工程，全面兴起新一轮干事创业热潮。

今年政府工作的总体要求是：高举习近平新时代中国特色社会主义思想伟大旗帜，全面贯彻党的十九大和十九届二中、三中全会精神，认真落实党中央国务院、省委省政府和市委各项决策部署，统筹推进“五位一体”总体布局和“四个全面”战略布局，坚定不移沿着“八八战略”指引的路子走下去，抢抓机遇、清醒有为，奋力续写好温州这部创业创新史，聚焦聚力改革开放、动能转换、协调发展、文化兴盛、固本强基，系统构建五大发展生态，加快建设国际时尚智城，努力再造改革开放新优势、再创高质量发展新辉煌、打造坚实“铁三角”，争当全省“两个高水平”建设的排头兵，以优异成绩向改革开放40周年献礼。

全市经济社会发展的主要预期目标是：地区生产总值增长7.5%；财政收入增长与经济增长基本同步，财政总收入超800亿元、一般公共预算收入达500亿元；固定资产投资增长10%；城乡居民人均可支配收入分别增长8%和9%；研发经费支出占地区生产总值比重达2.1%；单位生产总值能耗和污染排放量确保完成省下达目标。

做好今年政府工作，必须把握好以下四个方面：

(一)必须牢固树立高质量发展意识。高质量发展是能够很好满足人民日益增长的美好生活需要的发展，是体现新发展理念的发展，是创新成为第一动力、协调成为内生特点、绿色成为普遍形态、开放成为必由之路、共享成为根本目的的发展，是引领温州未来的发展。当前，温州正处在转型升级、爬坡过坎的重要关口，我们必须牢牢把握高质量发展的内涵和要求，深化以“破”“立”“降”为重点的供给侧结构性改革，全面实施“新动能培育21条”，大力推动传统产业高端化、战略性新兴产业和高成长型生产性服务业协同发展，加快建设现代化经济体系，促进经济社会发展主要指标晋等升位，不断增强区域经济创新力和竞争力。

(二)必须坚定扛起为新时代改革开放探路的使命。以纪念改革开放40周年为契机，实施“改革攻坚突破年”行动，解放思想、卸下包袱，不为声名所累、不为路径所惑、不为模式所困，以新理念引领温州新发展、以新改革增添温州新动力、以新开放拓展发展新空间，重整旗鼓再出发，努力再现温州风向标式的辉煌。大力弘扬温州人敢为人先的创新精神、敢闯天下的开拓精神，以舍我其谁的情怀和自我革新的勇气，加速打造新时代民营经济“两个健康”发展先行区，争当新时代中国民营经济发展的领跑者。坚持以开放促改革促发展，积极对接“一带一路”，深度参与国际产能合作，加快城市国际化步伐，着力构筑全面开放发展新格局。

(三)必须落实以人民为中心的发展思想。坚持把人民对美好生活的向往作为奋斗目标，适应社会主要矛盾变化，着眼于解决城乡之间、区域之间、群众收入之间的不平衡和经济转型、城市发育、社会建设的不充分等问题，抓重点、补短板、强弱项，坚决打好防范化解重大风险、精准脱贫、污染防治“三大攻坚战”，努力实现更平衡更充分的发展。坚持在发展中补齐民生短板、促进社会公平正义，从解决群众最盼、最急、最忧、最怨的问题入手，出实招、办实事、求实效，把群众最关切最烦心的事一件一件解决好，使全市人民的生活随着温州的发展一年比一年更好。

(四)必须强化有为政府的果敢担当。坚持有效市场与有为政府相结合，按照强谋划、强执行的要求，以提高行政质量、行政效率和政府公信力为目标，加快建设人民满意的服务型政府。坚持依法履职，坚持有为担当，坚持干字当头，集中精力推进一批牵一发动全身的大事、攻克一批跨越关口的难事，多做打基础利长远的工作，创造经得起实践、人民和历史检验的业绩。

根据上述目标要求，今年重点做好以下八方面工作：

(一)以更大勇气更大力度全面深化改革开放，努力增创体制机制新优势。

将“最多跑一次”改革进行到底。优化“四个一”创新工程和“一窗受理、集成服务”，年底前所有民生事项和企业事项实现一次办结，其中80%以上开通网上办理，50%以上的民生事项实现一证通办，大幅提升“零上门”办结事项比重。加快项目审批速度，10月底前实现一般企业投资项目开工前审批全流程最多跑一次、最多100天，力争竣工验收最多跑一次、最多30天。推进“多证合一”“证照分离”，积极探索“照后减证”。投用温州市民中心。依托政务服务网，推广移动办事终端，将“最多跑一次”全面延伸到镇街和村(社区)。全面放开涉批中介服务市场，开展涉批中介机构清理整顿专项行动。大力推行“双随机、一公开”监管，切实提高事中事后监管水平。健全统一的政务咨询投诉平台，做到“一个号码”响应群众诉求。

推动十大重点领域改革取得实效。以系统思维推进金融综合改革、农村产权制度改革、市场取向改革、国资国企改革、科技体制改革、工程领域制度改革、全域统筹机制改革、住房市场改革、生态文明体制改革、容错纠错制度改革等十大重点改革，加快形成点上突破、面上协同的集成改革亮丽风景。全面实施金融综合改革2.0版，壮大地方金融板块，发展普惠金融，优化多层次融资结构，增强金融服务实体经济能力，新增融资规模1200亿元。坚决打好防范化解重大风险攻坚战，开展政府隐性债务清理化解工作，持续推进“两链”风险化解，依法严厉打击逃废债，银行贷款不良率力争控制在1.8%以内。启动新时代民营经济“两个健康”发展先行区建设。健全企业合法权益依法保护机制，着力激发各类市场主体活力。加快推行企业对标竞价的“标准地”制度，30%的新批工业用地按“标准地”制度供地，促进生产要素向优质高效领域流动。深化国有平台、国企内部整合重组，推动国有企业完善现代企业制度，启动国有资本运营公司试点，促进国有资本做强做优。完善全市一盘棋统筹、一体化推进的工作协调机制。深化小城镇综合改革，推动龙港国家新型城镇化综合试点取得突破。抓好国家海域综合管理创新试点和国家海洋经济示范区建设，完善生态用海、科学管海机制，优化存量海域资源配置，提高围填海区域利用效率。

以“一带一路”为统领构建对外开放新格局。充分挖掘海内外温州人网络资源，组织开展“一带一路”温州行动，积极参与捷克站、迪拜站建设，提升发展境外经贸合作区，加快布局跨境电商公共海外仓，谋划建设国别产业园。加强海外侨团服务管理和涉外风险防范。推进贸易和投资便利化改革，争取设立跨境电子商务综合试验区，加快建设国际贸易“单一窗口”。做大做强保税物流中心。主动对接首届中国国际进口博览会，争取国家市场采购贸易方式试点，实现货物贸易进出口增长与全省同步、服务贸易进出口增长10%左右。积极参与长江经济带等国家战略，主动融入区域一体化发展。继续抓好对口支援工作。

以一流的营商环境加速项目引进来。坚持刀刃向内，以刮骨疗毒的决心和勇气，开展“营商环境提升年”行动。严格实施“深化改革优环境11条”“执纪监督治怠政9条”，成立营商环境服务监督局，设立企业综合服务平台。实行涉企税收柔性服务，强化涉企政策刚性兑现，建立有求必应的营商专员制，着力打造不诉不查的涉企执法环境，让海内外广大客商“投资温州、办事无忧”。精心组织招商引资“生命线工程”，以“重大项目谋划盯引”“市县委书记和市县长项目工程”为载体，瞄准温商领头雁企业重大技术升级项目、世界500强区域战略布局项目、战略性新兴产业项目、现代服务业项目及重大产业融合项目开展精准招商，力争引进10亿元以上重大产业项目20个，其中50亿元以上2个，民间投资增长10%以上。积极培育壮大温企跨国经营主体，支持企业实施以核心技术、研发设计团队和知名品牌为重点的境内外并购。把利用外资与调整优化产业结构结合起来，创新投资促进机制，争取实际利用外资5亿美元。

(二)加速新旧动能转换，积极构建现代化经济体系。

大力推动传统产业高端化发展。实施传统产业改造提升行动计划，引导企业运用新技术、新业态、新模式实现网络化协同、个性化定制、柔性化生产、服务化延伸，积极争创“中国制造2025”国家级示范区。规划建设产业创新服务综合体10个以上，新增省级两化融合示范区1个、行业云应用服务平台2个以上，培育两化融合示范试点企业30家、“上云”企业1.5万家。推动传统产业提高智能化和工艺装备水平，实施“百项百亿”重点技改项目和百项发明专利产业化项目，鼓励建设“无人工厂”“无人车间”，力争新增机器人应用1200台，工业投资增长8%，全员劳动生产率增长8%。鼓励制造企业与知名工业设计机构开展深度合作，加快时尚智造设计中心建设。实施小微园质效提升行动，提升建设品质，把好入园门槛，合理控制价格，建成投用小微园300万平方米以上。

培育壮大战略性新兴产业。认真落实全省数字经济“一号工程”战略部署，大力发展以数字经济为核心的新产业，加强与阿里巴巴集团的战略合作。对接全省大湾区战略，加快打造浙东南临港产业带，积极构建温州湾区产业协作体系。按照每个重点产业平台1-2个主攻方向的要求，突出引进和布局新一代信息技术、生物医药、新材料、激光光电、清洁能源等新兴产业，加快苍南绿色能源、正泰物联网传感器产业园等项目建设，推动战略性新兴产业增加值增速高于规上工业增加值平均增速5个百分点以上。深度谋划集群战略，启动电气、时尚、新能源网联汽车和智能装备四大千亿级产业集群打造计划。

提升发展现代服务业。大力扶持发展信息服务、科技服务、检验检测、法律会计、供应链管理等生产性服务业。鼓励制造业企业分离发展服务业，引导实施10个总集成总承包重点项目，推动生产性服务业占服务业比重上升2个百分点。提速建设滨江商务区金融集聚区，加快发展现代金融、总部经济，全市金融业增加值增速提高2.5个百分点。提升发展健康养生、教育培训、物业管理等生活性服务业，推动商品市场线上线下融合发展和功能形态升级。完善旅游管理体制，做强旅游投资集团，抓好高等级景区品质提升和旅游风情小镇建设，提升雁荡山、江心屿等景区开发管理水平，支持楠溪江、刘伯温故里等创建5A级景区，支持泰顺创建廊桥-氡泉国家级旅游度假区，加快打造“中国著名休闲度假旅游目的地”。推动永嘉、文成、苍南、平阳、洞头发展全域旅游，全市旅游总人次、总收入均增长15%。

支持企业做强做大。实施领军企业、高成长型企业“培优计划”，重点支持200家企业通过科技创新、产业链整合、资本运作等方式，推动规模和效益“三年翻番”。实施大企业(集团)培育工程，推动形成一批具有核心竞争力的企业群体。实施百企上市培育计划，力争新增上市报会企业15家、新三板挂牌企业10家、股份公司100家。新增省“隐形冠军”企业3家、工业企业“小升规”500家、服务业企业“下转上”150家。做好全国第四次经济普查工作。全面落实实体经济降本减负政策，力争为企业减负100亿元。实施“青蓝接力”计划，打造一支具有全球视野、创新能力和互联网思维的新型企业家队伍。

(三)高水平实施创新驱动发展战略，倾力打造国家自主创新示范区。

完善区域创新发展布局。全力建设国家自主创新示范区，聚焦核心区域，突出民营经济特色，构建完整创新创业生态，全面提升温州民营经济自主创新能力。加快建设浙南科技城，在先行区初步构建示范型产城融合样板和区域性协同创新中心，支柱型产业项目招引取得关键性突破，高新技术产业增加值增长10%，推动温州高新区形成更加清晰的高新产业发展导向。按照发展高端产业和产业高端化的要求，加快瓯江口产业集聚区、浙南产业集聚区产城融合发展，着力打造成为全面践行新发展理念的高端平台，力争两大产业集聚区产业投资增速高于全市平均水平8个百分点以上。鼓励国内外高校院所和知名企业来温建设新型研发机构，抓好生命健康小镇等省市特色小镇培育创建。深入推进海峡两岸(温州)民营经济创新发展示范区试点工作。高标准规划建设高教新区，提升发展大学科技园。推进科技成果权益管理改革，充分发挥好温州医科大学、温州大学等高校的创新源头作用。

激发社会创新创业活力。深入实施规上工业企业科技创新“三清零”行动，争取规上企业科技活动覆盖率和研发机构设置率提高到60%。建立企业研发“后补助”制度。落实科技企业“双倍增”计划，新增高新技术企业180家、科技型中小企业1500家，确保高新技术产品产值占工业总产值、高新技术产业增加值占工业增加值比重“双提升”。开展“标准领跑者”“对标达标”行动，争取规上企业主导产品采标率超70%，新增各类标准100项，新增“浙江制造”品牌认证9家，培育“品字标”企业40家。加大知识产权保护力度，积极申报创建国家知识产权强市，新增发明专利授权2600件。

优化创新要素支撑体系。研究制定更加积极、开放、有效的人才新政，着力打造一流的引才留才用才环境，吸引高层次人才来温创新创业。建立专家和留学归国人员服务中心，抓好“千人计划”温州产业园建设，全市新增“千人计划”“万人计划”30人，培育引进领军型团队和项目30个。重视建设高技能人才队伍。高质量建设双创示范基地，办好创博会和创客文化节，优化提升创业服务，推动“四新”经济发展。进一步发挥政府产业基金、科创基金作用，加快构建产业投资基金体系，切实提高基金使用绩效。加大面向创新型企业的担保增信、风险补偿、贷款贴息等政策支持力度，促进天使投资、股权投资基金在温集聚，扶持本土创新项目和初创期、种子期项目。深化要素市场化配置改革，完善“亩均论英雄”评价管理制度，集中开展“批而未供”“供而未用”土地清理专项行动，全年消化“批而未供”土地2万亩，处置“供而未用”土地4万亩。强化对平台用地进度和产出效益的评估考核，全面整治亩均税收1万元以下低小散企业，大力破除资源要素无效供给，积极创建全国国土资源节约集约模范市。

(四)高品质推进都市区建设，实现“城市让生活更美好”。

加速城市精彩蝶变。以决战决胜姿态坚决打赢城中村改造攻坚战，完成49个行政村、60个区块(旧片区)改造，签约4.5万户，其中市区完成30个行政村、18个区块(旧片区)改造，签约2.68万户，确保6月底前市区核心区内所有特殊建筑处置到位、2017年前启动的城中村改造项目“清零清场”。坚持功能性项目先行，统筹地上地下空间利用，实施中心城区“大建大美”项目552个、建成169个。突出补齐公共服务和市政设施短板，新开工教育项目23个，确保秋季开学前投用小学、幼儿园29所。加力加速推进“两线三片”开发建设，确保国庆节前，瓯江南岸景观改造提升一期和亮化夜游工程建成试用，温瑞塘河完成沿河滨水公园和亮化夜游工程建设，五马历史文化街区展现新貌，中央绿轴南段全面贯通、北延整体提升，三垟城市湿地公园实现初步开园。加快建设瓯海高铁新城。持续抓好海绵城市和地下综合管廊规划建设。高标准实施市区15条道路综合整治，联动推进背街小巷整治和架空管线上改下，为市民出行创造更美环境。编制都市区规划，积极推动县域经济向都市经济转型，加快建设各县(市)城市重点区块。

着力构建“外快内畅”交通网络。对接全省大通道建设，完善城市综合交通规划体系，推动瓯江两岸、温瑞平原、温州南部等区域综合交通一体化发展，确保交通投资增长15%以上。全线推进杭温高铁一期，同步建设温州北站，加快东部交通枢纽及线网规划建设。围绕“市域1小时交通圈”目标，加快推进市域铁路S2线、绕城高速北线二期、龙丽温高速瑞文段、溧宁高速文泰段、瓯江北口大桥建设，开工溧宁高速文景段，建成乐清湾港区铁路支线、甬台温复线南塘至黄华段等工程。大力实施城区交通内畅工程，市域铁路S1线建成试运行，温瑞大道二期拓宽工程确保上半年开工、年底基本建成。持续推进打卡口、接断路，畅通道路微循环。推动城区快速公交成环成网，建成BRT3号线、4号线，开工5号线、6号线，加密和优化市区对外辐射常规公交线路，初步构建轨道交通与常规公交换乘系统。新建“四好”农村公路150公里，提升改造500公里，实施安保工程700公里。加快市域铁路S3线、“四连高速”、大罗山隧道及连接线、三都岭隧道拓宽改造等项目前期，积极推动温武吉铁路、温福铁路、轨道交通M线等前期工作。

提高城市精细化管理水平。制定实施智慧城市建设三年行动计划。规划建设5G移动通信网络，建立通信、物联等信息基础设施资源共享机制，促进城市公共服务设施互联互通。建设“城市数据大脑”，推动智慧交通系统升级、智慧城管功能扩容。建设智慧停车云平台，有序推动机关、企事业单位停车泊位向社会错时开放。引导共享单车、城市配送等消费新业态规范发展。深化城市管理治乱专项行动，集中梳理一批城市管理的痛点作为执法重点，以最硬的措施向各类顽疾乱象宣战。健全市政设施养护长效机制。深入推进“三改一拆”，强化违法建筑常态化管控，创建基本无违建县(市、区)3个，基本无违建乡镇(街道)覆盖面达到70%。推动乡镇(街道)综合行政执法全覆盖，形成重心下移、责权一致、高效有序的城市管理机制。

(五)高起点实施乡村振兴战略，推动城乡融合互动发展。

加快农业农村现代化建设。按照“产业兴旺、生态宜居、乡风文明、治理有效、生活富裕”总要求，大力实施乡村振兴“六千六万”行动，着力打造高效生态开放的新农业，培育高素质有组织的新农民，建设高颜值和美的新农村。推动产业集聚，建设省级现代农业园区2个、特色农业强镇4个，打造一批田园综合体、乡村民宿带、森林康养基地，促进一二三产融合发展。加快推进绿色兴农、质量兴农、科技兴农，开展早茶、铁皮石斛等农产品区域公共品牌创建，建成绿色农业科技示范基地200个，规模以上主体全面纳入追溯平台管理。实施农业龙头企业“3365”培育计划，新增市级农业龙头企业、示范性家庭农场各50家。全面落实粮食安全责任制。启动实施11个全域土地综合整治工程，垦造耕地1万亩。深化美丽乡村建设，完成39个小城镇环境综合整治，创成美丽乡村样板村20个、A级景区村200个，建成乡村振兴(美丽乡村)示范带11条。统筹推进乡村治理和农村文化建设，大力开展移风易俗行动，打造善治示范村200个、文化礼堂706个。深化“三位一体”新型农村合作体系建设，推动农业社会化服务升级。加强城乡电商服务点和城乡连锁便利店建设。深入推进农村产权制度改革，开展“共享农房”试点，探索宅基地所有权、资格权、使用权“三权分置”，激活农民财产权。健全城乡融合发展机制，推动资本、技术、人才下乡和绿色优质农产品进城，全面构筑新型城乡关系。

扎实推进西部生态休闲产业带建设。对接全省大花园战略，聚焦聚力11个生态休闲重点区块，全面实施生态大保护、休闲旅游品质、生态休闲农业发展、美丽乡村升级、交通发展基础五大工程，努力把我市西部建设成为环境美、产业优、交通畅、百姓富的全国“两山”转化样板区、中国生态休闲产业创新区和乡村振兴重点区。积极开展重要生态系统保护和修复行动，进一步提升西部生态环境质量。系统构建西部绿色产业体系，谋划落地173个产业项目。推进西部交通网、旅游通道网、慢游道路网“三网”共建，加快G104、G228等一批国道省道联网项目建设，改造提升雁楠、平苍文泰等旅游交通环线，创建美丽生态示范路11条，建成美丽经济交通走廊860公里。

坚决打好低收入百姓增收攻坚战。完善精准帮扶机制，深入实施低收入农户收入倍增计划，确保有劳动能力的低收入农户人均收入增长15%以上，巩固家庭人均年收入4600元以下贫困现象消除成果。努力解决因病致贫返贫和农村基本住房安全保障问题，基本完成农村困难家庭危房改造。因地制宜开展生态大搬迁行动，完成地质灾害避让搬迁、下山移民、低收入农户异地搬迁等2.9万人。建立村级集体经济“消薄”反弹防滑机制，确保所有村集体经济年总收入超10万元、年经营性收入不低于5万元。深化山海协作机制，加快提高少数民族地区、革命老区、偏远山区和库区海岛基本公共服务水平。

(六)高标准建设生态文明，提升“美丽温州”新形象。

坚决打好污染防治攻坚战。全面落实中央环保督察、国家海洋督察整改要求，严格环保执法和海洋监管，着力解决好突出问题。巩固“五水共治”和剿劣攻坚成果，严格落实河长制、湖长制，开展美丽河道创建，确保污水治理不反弹。完善城区污水处理设施规划布局，建成投用中心片污水处理厂迁扩建工程，启动5座城镇污水处理厂清洁排放技术改造，关停整合小型污水处理设施。实施新一轮污水管网排查整治提升三年行动，新建改造城镇污水配套管网400公里，市区6月底前完成99项截污纳管工程，下半年再完成98项。建立市区两级排水管网建管维养长效机制，创建“污水零直排区”11个。持续推进重点流域综合治理，抓好17个“百项千亿防洪排涝工程”，开工建设温瑞平原东片、西片排涝等项目，加快鳌江干流治理水头段防洪工程建设，基本建成瑞安飞云江治理一期、文成飞云江治理二期。深化瓯江引水工程前期工作。坚决打赢重污染行业整治收官战，全面完成1850家企业整治任务。深入开展大气污染防治，严格执行工业废气清洁排放标准，实施挥发性有机物治理专项行动，完成重点行业废气清洁排放技改项目10个，着力解决工业臭味扰民问题。加强土壤污染治理修复，抓好农业面源污染防治。统筹推进垃圾处理设施建设，建立城乡垃圾分类投放、分类收集、分类运输、分类处理系统。推进“厕所革命”，加强城乡厕所规划建设管理。

加强生态保护和建设。统筹推进山水林田湖生态保护和修复，加强生态主体功能区建设，确保生态环境投资增长12%以上。推进要素生态化配置综合改革，建立绿色发展财力支撑、出境水质互动机制，编制实施产业准入负面清单，全面推行生态环境损害赔偿制度。突出抓好珊溪、泽雅、楠溪江等水源地保护，完善市场化、多元化生态补偿机制。加强大罗山保护、建设和管理。扎实做好国家园林城市复评工作，继续抓好城乡公园、绿道和慢行系统建设，推动“绿满温州”。启动实施海洋生态建设三年行动，联动整治近岸海域污染，加快建设生态海洋。深化国家低碳城市试点，加快推进能源绿色化改造和电能替代，积极推广屋顶光伏发电，提高清洁能源消费比重。

(七)高质量保障和改善民生，不断增进民生福祉。

优先发展教育事业。全面落实加快基础教育发展若干意见，积极推进教育现代化县(市、区)创建，新改扩建中小学校208所，进一步促进教育公平，缩小城乡校际办学差距。实施学前教育补短提升工程，普惠性幼儿园占比提高到85%。在育儿机构推广远程视频应用，让家长放心安心。开展消除“大班额”和班额控制专项行动。探索实行学后托管服务。推进普通高中优质均衡发展，进一步提高全市高考一段录取率。积极引进国内外一流大学来温合作办学，推动在温高校办出特色、更好服务地方。加快8所职校迁扩建工程，建设一批职业教育精品特色专业。实施瓯越教育人才培养计划，完善教师队伍激励机制。健全市区教育一体化发展机制和初高中办学体制，推进管办评分离和中小学教师“县管校聘”改革。深化民办教育综合改革。

加快健康温州建设。推动优质医疗与康养服务相结合，着力打造区域医疗康养中心城市。深化医药卫生体制改革，加快建立现代医院管理制度。深入实施“双下沉、两提升”，巩固完善分级诊疗制度，推进县域医疗共同体建设，进一步提升县域和基层就诊率。加强基层医疗卫生服务体系和全科医生队伍建设，家庭责任医生规范签约率达35%，乡镇卫生院(社区卫生服务中心)标准化率达到95%以上。健全疾病预防控制体系。支持中医药产业发展。鼓励社会力量优先投向医疗资源稀缺领域。完善与两孩相适应的人口计生服务管理制度，加快妇幼保健机构建设，加大“两非”打击力度。加强群众体育设施建设，开工温州奥体中心主体育场，做好2022年杭州亚运会温州分赛区筹备工作，推进社会力量办体育国家试点，加快打造国家运动健康城市。

加快文化温州建设。深入践行社会主义核心价值观，持续开展“温州好人”“最美温州人”“温州工匠”等活动，大力弘扬城市人文精神，唱响主旋律。加强温州题材文艺精品创作，讲好新时代温州故事。拓展城市书房、百姓书屋、共享书巢等阅读阵地，开展墨香校园、墨香家庭系列创建，打造“书香温州”“墨香温州”。深入实施文化惠民工程，基层公共文化综合服务中心实现社区全覆盖，努力创建国家公共文化服务体系示范区。开展“温州学”研究，充分挖掘刘基、叶适、孙诒让、郑振铎、南怀瑾等文化名人资源，推动瓯越文化元素融入城市建设和市民生活，切实增强对温州文化的认同与自信。建成“世界温州人家园”，着力打造情感地标、精神家园。

解决好群众住有所居问题。坚持“房子是用来住的、不是用来炒的”定位，加快建立“多主体供给、多渠道保障、租购并举”的住房制度。完善做地、储备、出让联动机制，科学把握供地结构和节奏，合理引导市场预期和购房行为，促进房地产市场平稳健康发展。深入开展省级住房租赁试点，多渠道增加租赁住房供应。统筹推进异地安置和货币化安置，加快安置房及配套设施建设，市区新开工保障性安居工程4.2万套、625万平方米，竣工1.4万套、201万平方米。全面实施天然气入户三年行动计划，逐步减少城区液化气供应站，城区管网覆盖率达到80%。

加强就业和社会保障工作。坚持以创业带动就业，积极推进大学生驿站和创业工坊建设，新增在温就业大学生3.6万人。全面实施全民参保计划，户籍人口基本养老保险参保率达到90%，基本医疗保险参保率稳定在98%以上。完善城乡社会救助体系，统一城乡最低生活保障标准。深入开展全国养老服务业综合改革、全国医养结合试点，规划建设一批养老机构和示范型居家养老(服务)中心，新增养老机构床位5000张以上，启动长期护理保险制度试点。深入实施残疾人康复工程，促进残疾人多渠道就业。支持工青妇等人民团体建设，进一步做好老龄、慈善、红十字、外事、人防、气象等工作。

(八)加强和创新社会治理，切实维护社会和谐稳定。

全面加强平安温州建设。坚决守住治危防患、防汛防台、安全生产和社会稳定“四条红线”。大力开展扫黑除恶专项斗争，高水平推进“雪亮工程”，依法打击和惩治黄赌毒黑拐骗等违法犯罪活动，创建全国禁毒示范城市。深入实施“治危拆违”“除险安居”专项行动，全面消除涉及公共安全的城乡危旧房和地质灾害重大隐患。深化安全生产领域改革，推进全国安全生产宣传教育“七进”活动试点，铁腕整治消防和道路交通、危化品、渔业船舶、特种设备等重点领域安全隐患，促进全市安全生产形势根本性好转。高度重视“舌尖上的安全”，开展食品药品安全隐患大整治行动，力争创成5个省级食品安全示范县(市、区)。

提升社会治理水平。创新发展新时代“枫桥经验”，切实发挥好基层治理“四个平台”和全科网格的作用，构建完善“一三五”应急处置机制，巩固提升“一体两翼”基层社会治理体系。加强社会矛盾排查预警，完善初信初访化解和人民调解机制，加大信访积案和历史遗留问题化解力度。加快构建多元化基层社会治理模式，加强社工服务机构和队伍建设，发挥好社会组织在社会管理和公共服务中的积极作用。稳步推进撤村建居和行政村撤并。深化“7431”网络治理模式，打造清朗网络空间。深入实施“六大诚信建设工程”，健全信用奖惩机制。深化“温州无欠薪”创建行动，着力构建和谐劳动关系。认真开展“七五”普法，完善司法救助和法律援助制度。依法加强宗教事务管理。

促进军民深度融合发展。支持国防和军队建设，提升国防动员和后备力量建设水平，争创省级双拥模范城“六连冠”。积极引进军民融合技术和项目，打造一批“军转民”“民参军”温州品牌。

各位代表，增进民生福祉是发展的根本目的，是政府工作的出发点和落脚点。按照民生实事项目人大代表票决制的有关要求，在广泛征求意见的基础上，我们梳理了12件民生实事候选项目，提请本次大会票决。对票决选出的10件实事，我们将强化组织领导、严格责任考核、狠抓工作落实、主动接受监督，确保把实事办好、把好事办实。

三、深化政府自身改革和建设

面对繁重艰巨的改革发展任务，全体政府工作人员要深入贯彻落实新发展理念，按照省政府提出的“两强三提高”要求，切实加强政府自身改革和建设，促进政府治理能力现代化，不断开创政府各项工作新局面。

(一)依靠学习走向未来，提高科学履职能力。深入学习贯彻党的十九大精神，自觉以习近平新时代中国特色社会主义思想武装头脑，牢固树立“四个意识”，高标准严要求开展“不忘初心、牢记使命”主题教育，在思想上政治上行动上同以习近平同志为核心的党中央保持高度一致。把深度学习作为终身本领，把业务学习作为看家本领，完善和落实政府各项学习制度，在学习中提高本领，在实践中锤炼本领，不断提高研究谋划、解决问题、狠抓落实的能力，努力把学习调研成果转化为推动温州高质量发展的有效举措。

(二)依靠制度畅通政令，提升政府执行力。贯彻党和国家机构改革决策部署，全面落实地方政府机构改革各项任务，加强和完善政府经济调节、市场监管、社会管理、公共服务、生态环境保护职能，形成职责明确、依法行政的政府治理体系。加强对重大决策部署落实执行情况的督促检查，完善政府工作"134"责任机制，不断优化强执行的制度环境。坚决治理为官不为、懒政怠政，进一步提振攻坚克难的锐气，让奋发有为、敢于担当成为政府公务人员干事履职的新常态。

(三)依靠法治依法施政，增强政府公信力。弘扬宪法精神，加强宪法学习，增强宪法意识，全面贯彻宪法修正案和《监察法》，把政府活动全面纳入法治轨道。认真执行市人大及其常委会的决议决定，坚持重大事项报告制度。自觉接受政协民主监督，主动听取民主党派、人民团体和各界人士的意见建议。高质量办好议案、建议和提案。科学编制政府立法计划，积极配合人大立法。坚持科学、民主、依法决策，探索开展重大行政决策目录化管理。深化行政复议体制改革。构建统一行政执法信息平台，切实做到严格规范公正文明执法，防止用权任性。坚持诚信施政，各级政府要信守承诺，决不“新官不理旧账”。

(四)依靠科技支撑善政，切实提高行政效率。着力推动政府数字化转型，健全政府数据资源管理制度，强化政府数据资源的统一规划、统筹使用，逐步向社会开放政府信息资源。深入实施互联网+政务服务，推广应用电子签名、数字证书、电子印章，提高跨层级、跨部门政务协同效率。实施政务大数据应用示范工程，积极利用物联网、大数据提高政府在经济调节、市场监管、社会管理等领域的快速反应和精准治理能力。

(五)依靠严治正风肃纪，树立政府良好形象。严格落实全面从严治党主体责任，按照建设清廉温州的要求，全面加强政府系统廉政建设，努力营造风清气正的政治生态。高标准落实中央八项规定实施细则精神，坚决杜绝“四风”问题隐形变异、反弹回潮。围绕权力运行重点领域、关键环节，深入探索廉政建设与业务工作融合机制，严厉整治发生在群众身边的腐败问题和破坏营商环境的作风问题。坚持政府过紧日子，切实规范公务接待、因公出国(境)管理，从严控制“三公”经费支出，腾出更多的财力用于民生事业。

各位代表：时序交替、梦想前行。改革开放40年来，我们依靠敢为人先、特别能创业特别能创新的温州人精神，闯出了一条具有鲜明特色的发展之路。站在新的历史起点上，我们一定要以更加强烈的使命担当、更加开放自信的胸襟气度，锚定高质量发展新目标，在新时代新征程上再次扬帆起航，不忘初心，逐梦远行。让我们紧密团结在以习近平同志为核心的党中央周围，在中共温州市委的坚强领导下，紧紧依靠全市人民，团结拼搏、锐意进取，系统构建五大发展生态，加快建设国际时尚智城，为再造改革开放新优势、再创高质量发展新辉煌、打造坚实“铁三角”、争当全省“两个高水平”建设的排头兵而努力奋斗！
